# Assessing the Impact of Policy Changes in the Icelandic Cod Fishery Using a Hybrid Simulation Model

**DOI:** 10.1155/2014/707943

**Published:** 2014-02-24

**Authors:** Sigríður Sigurðardóttir, Björn Johansson, Sveinn Margeirsson, Jónas R. Viðarsson

**Affiliations:** ^1^Faculty of Industrial Engineering, Mechanical Engineering and Computer Science, University of Iceland, Hjarðarhagi 2-6, 107 Reykjavík, Iceland; ^2^Matís, Icelandic Food and Biotechnology Research, Vínlandsleið 12, 113 Reykjavík, Iceland; ^3^Product and Production Development, Chalmers University of Technology, 412 96 Gothenburg, Sweden

## Abstract

Most of the Icelandic cod is caught in bottom trawlers or longliners. These two fishing methods are fundamentally different and have different economic, environmental, and even social effects. In this paper we present a hybrid-simulation framework to assess the impact of changing the ratio between cod quota allocated to vessels with longlines and vessels with bottom trawls. It makes use of conventional bioeconomic models and discrete event modelling and provides a framework for simulating life cycle assessment (LCA) for a cod fishery. The model consists of two submodels, a system dynamics model describing the biological aspect of the fishery and a discrete event model for fishing activities. The model was run multiple times for different quota allocation scenarios and results are presented where different scenarios are presented in the three dimensions of sustainability: environmental, social, and economic. The optimal allocation strategy depends on weighing the three different factors. The results were encouraging first-steps towards a useful modelling method but the study would benefit greatly from better data on fishing activities.

## 1. Introduction

Our planet has a limited amount or resources available, and in today's global market, several of the resources are extracted faster than they are replenished. In fact only two countries in the world today do have positive regrowth in comparison with the extraction in terms of CO_2_ [1]. While the population on the planet is increasing, a question on how to sustain fair living conditions according to Maslow's Theory of Human Motivation [2] when it comes to food supply is one of the main challenges according to the UN Food and Agriculture Organization [3].

Fish and fishery products are an important source of protein for human consumption. In 2009, 16.6% of the world population's intake of animal protein came from fish and 6.5% of all protein consumption and globally fish provides 3 billion people with almost 20% of their intake of animal protein [4].

Despite the current knowledge in fisheries science, many of the world's fishing nations still face problems in managing their fisheries. FAO estimates that almost 30% of all fish stocks are overexploited, thus producing lower yields than they potentially could and are in need of strict management plans to restore full productivity and stocks [4].

In the case study presented in this paper, we look at how a simulation model can be used to assess a fishery in terms of the impact from management decisions. We choose the Icelandic cod fishery as it is well documented and data is easily accessible.

### 1.1. Icelandic Cod Fisheries

**Figure 1:**
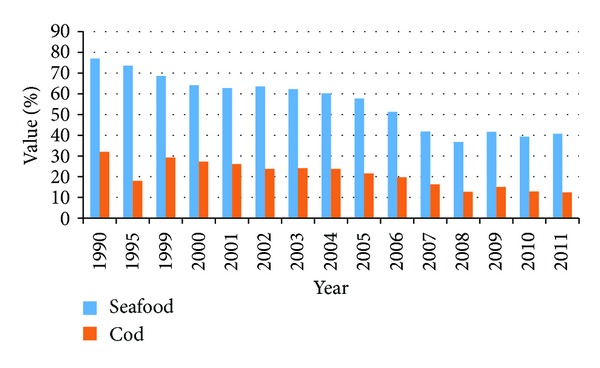
Ratio of seafood of total value of exports and ratio of cod in total value of seafood during 1990–2011.

Historically, the seafood sector has been the single most important industry in the Icelandic economy with cod fishery as its backbone. Even though other industries have been growing larger during the years, the seafood industry is still considered the most important one. National accounts show that in the year 2011, exported seafood accounted for more than 40% of total exports, with cod explaining more than 12% [5].  Figure 1 shows value of exported seafood as a percentage of total exports. Moreover, it has been estimated that the contribution of the fisheries sector and related industries, or the so-called fisheries cluster, to the GDP in the year 2010 is 26% [6]. 

In the 1980's, recruitments of cod began to reduce drastically while at the same time fishing effort remained higher than recommended by the Marine Research Institute. Stock levels of cod reached a critical level and to contain the situation a harvest control rule was developed to determine total allowable catch (TAC). In 1984, a comprehensive system of individual transferable quotas (ITQ) was introduced. In the beginning, quota was allocated based on vessel's previous catch records. The ITQ system resulted in an improved economic efficiency of the fisheries as well as biological viability [7, 8]. The merits of the quota system have however been heavily debated since its establishment due to the consolidation of quotas and the effect it has had on fisheries communities short of quota [9].

The Icelandic government has defined objectives with its fisheries management system which are to promote conservation and efficient utilisation of the exploitable marine stocks of the Icelandic fishing banks and thereby ensure stable employment and settlement throughout the country [10].

### 1.2. Purpose of Study

Considering the aforementioned objectives, new policies for managing the fisheries have to be assessed in the three dimensions of sustainability: economic, environmental, and social. In this paper we present a hybrid-simulation framework to assess the impact of changing the ratio between cod quota allocated to vessels with longlines and bottom trawls. It makes use of conventional bioeconomic models and discrete event modelling (DES) and provides a framework for simulating life cycle assessment (LCA) for a cod fishery. The model was constructed in AnyLogic and consists of two models: a system dynamics model describing the biological aspect of the fishery and a discrete event model for fishing activities.

### 1.3. Fisheries Models

Most simulation research in fisheries management is based on continuous multiparameter models. Tools that have been used previously for assisting in fisheries management are, for example, the multiparameter models FLR (Fisheries Library for R) and EcoSim. The FLR framework is a development effort directed towards the evaluation of fisheries management strategies [11]. Ecopath with EcoSim (EwE) is an ecosystem modelling software suite that allows for spatial and temporal modelling for exploring impact and placement of protected areas and policy assessment [12]. It is probably the best known ecosystem model and has been applied widely in fisheries around the world.

Atlantis [13] is a modelling framework developed to evaluate ecosystem based management strategies. It consists of a number of different linked modules: biophysical, industry and socioeconomic, and monitoring and assessment.

Many other modelling frameworks exist including Gadget [14] and BEMMFISH [15].

Most of these modelling frameworks allow for great details in the biological aspect of fisheries modelling but may lack overview in the three aforementioned dimensions of sustainability. The need for holistic modelling in fisheries has been emphasized [16]. System dynamics (SD) is a good tool for creating holistic models and understanding how things affect one another.

Dudley [16] has demonstrated the benefits of using SD for modelling fisheries and represented a framework that can be adapted to most fisheries. A number of system dynamics models in fisheries exist. A SD model of individual transferable quota system was constructed in order to differentiate ITQ from total allowable catch effects and identify areas where policy changes and management improvements may be most effective [17]. Other SD models include a model for the management of the Manila clam, a shellfish fishery in the Bay of Arcachon in France [18], a model of the management of the gooseneck barnacle in the marine reserve of Gaztelugtxe in Northern Spain [19], and a SD model of the Barents Sea capelin [20]. Finally, a hybrid model combining SD and agent based modelling has been constructed for understanding competition and cooperation between fishers [21].

### 1.4. LCA and Fisheries

Limited literature is available on LCA on fisheries and most of it comes from Scandinavia. Researchers at the Swedish Institute for Food and Biotechnology have contributed largely to this field. Ziegler and Hansson [22] assessed the emissions from fuel combusting in a Swedish cod fishery in terms of three scenarios reflecting different combinations of gear types, especially gillnet and trawls which are the most used gear types. Their results showed that gillnets show the lowest emissions compared to the other fishing gears and they emphasized the importance for high quality data on fuel consumption for future environmental studies. Ziegler et al. also carried out an LCA of frozen cod fillets and Ziegler [23] and Valentinsson performed an LCA of Norway lobster caught along the Swedish west coast [24]. The results from the frozen cod study which was carried out in 2003 revealed that, at that time, there was great room for improvement in terms of minimizing environmental impact from the cod fillets and, moreover, they highlighted the importance of good data for assessing the impact. In the more recent LCA of the Norway lobster published in 2008, the findings of Ziegler and Valentinsson were that the environmental impact from the fishery can be reduced considerably by shifting to creeling and selective trawls while still maintaining similar catch numbers. LCA of Danish fish products was carried out in 2006, with the focus on flatfish but also gave an overview of screenings of other fish species. There it was found that the fishing stage has the largest impact due to high fuel consumption and that large reductions can be made by switching to different fishing gear [25]. Finally, an LCA was carried out for Icelandic cod assessing in terms of two different fishing gears [26]. The results from that study were used in the study presented here.

### 1.5. Combining DES and LCA

Life cycle assessment (LCA) standardized by ISO 14040:2006 and 14044:2006 [27] is by far the most commonly used analysis method for evaluation of environmental footprint. LCA, however, holds drawbacks, which reduce its preciseness and limit its value for producing reliable results. The main associated problems with traditional LCA analyses are as follows [28].

- Using lumped parameters and site-independent models.
- Being static in nature and disregard of the dynamic behaviour of industrial and ecological systems.
- Focusing only on environmental considerations, not economic or social aspects.

Hence, it can be beneficial to complement LCA with other analysis tools, in order to effectively combine environmental and economic analysis. An example of such a combination is discrete event simulation (DES) and LCA. Various different examples of successful LCA-DES combinations have been carried out and presented before [29–32].

Most papers found focused on industrial process modelling with the LCA perspective describing models that are static compared to DES models. Examples of papers from different industrial areas are pharmaceutical intermediates [33], nitric acid plant, boron production [34], phenolic-resin manufacturing [35], and cement production [36]. By introducing environmental impact data for each event in a DES model we are be able to follow the environmental impact of the simulated system. Very much the same way as monetary units can be followed in this kind of system. Each event step in the model has environmental impact parameters. When the event is triggered, the environmental impact data will be put in play and update model output parameters. This enables prediction of the outcome from changes in reality more accurately, and also on a more detailed level if needed. Each product going through the system will have global warming (CO_2_ equivalents) and primary energy use (kWh), in addition to the normal parameters analyzed within DES, such as lead-time, utilization, and queue lengths.

Food production studies conducted using similar methodology as the one presented in this paper are rare. Some examples of initial cradle to gate studies where LCA data is used in a dynamic discrete event simulation model are:

- sausage production [37],
- juice production [38],
- yoghurt production [39].

## 2. Bottom Trawlers versus Longliners

**Figure 2:**
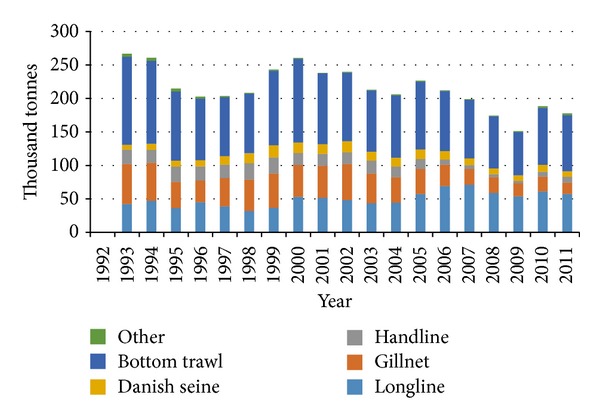
Total landings (thousand tonnes) of cod by fishing gear during 1993–2011.

Nowadays most of the Icelandic cod is captured in bottom trawls or with longlines. Use of gillnets used to be more widespread than of longlines but that has changed as Figure 2 confirms. In 2011 46% of the total allowable catch for cod was captured with bottom trawls and 32% with longlines [40], so around 78% of the total allowable catch is under consideration in this study.

Bottom trawls and longlines are very different fishing gears and have different economic and environmental impacts, and potentially social impacts which are harder to quantify and measure. Vessels with bottom trawls are significantly larger than the longliners.

### 2.1. Economic Impact

Data from operating accounts of fishing companies collected by Statistics Iceland reveal that the larger vessels are more economically viable [41]. During the years 2002–2007, the operation of smaller vessels was unstable, partly due to external factors such as high interest rates and strong exchange rate of the Icelandic krona [42].

### 2.2. Environmental Impact

When comparing bottom trawls with longliners in terms of minimising environmental impact, the longliner is a far better choice. In 2009, a life cycle assessment was applied to compare the environmental impact made when producing 1 kg of frozen cod caught with a bottom trawl on the one hand and a long line on the other. The conclusion from that study was that a trawled cod has a higher impact within all categories assessed such as climate change, respiratory organics/inorganics, ecotoxicity, acidification, and fossil fuel [26].

It has been reported that the distribution of corals around Iceland began to decline when bottom trawling was initiated [43]. The biggest drawback of longlines however is danger to marine animals such as sea birds that get stuck in the hooks of the longlines [44].

## 3. The Model

**Figure 3:**
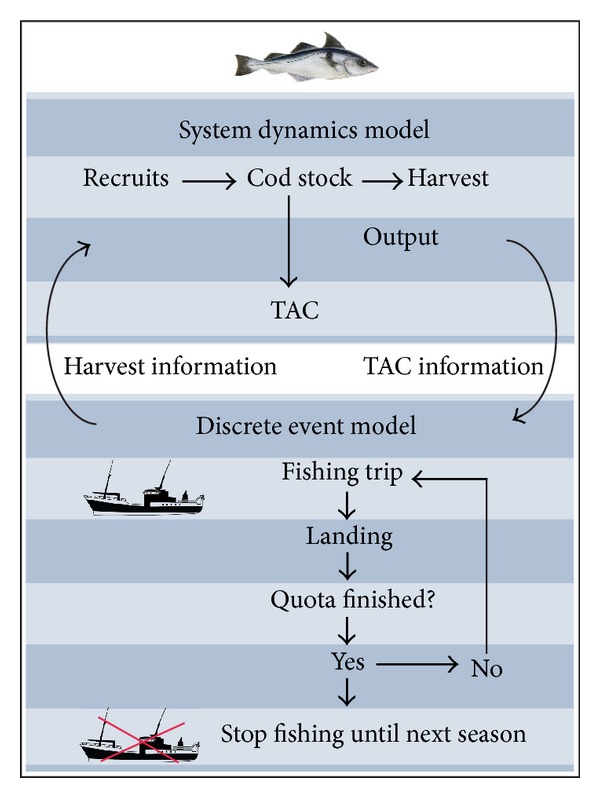
A diagram describing the hybrid-simulation model and the interaction between the SD model and the DE model.

A hybrid simulation model of the Icelandic cod fishery was constructed to assess the difference between the two fishing gears. The model consists of a system dynamics model that describes the growth of the cod stock. Fishing activities were simulated with a discrete event model.  Figure 3 shows a diagram of the model. The discrete event model simulates fishing trips of four different vessel types. Before a vessel starts a trip, it sends a query to the SD model to see if there is still catch quota available. If the total allowable catch is reached, no further fishing trips are planned until the TAC is updated for the following fishing year.

One of the key assumptions made in the model is that, every year, the vessels reach their catch quota. This is a valid assumption as the system holds a lot of fishing capacity and there is a demand for catch quotas and landing records confirm that they are always met [45].

### 3.1. A System Dynamics Model

The SD model describes the dynamics of the biological stock and provides the total allowable catch.

#### 3.1.1. Natural Biomass Growth Function

A simple biological model was applied to describe the biomass of cod. It accounts for no age-structure and the population dynamics are described with a logistic function [46]:

(1) $$\begin{array}{c} \dot{x}=rx\left(1-\frac{x}{K}\right), \end{array}$$

where *x* is the stock size of the fishable cod, *K* is the carrying capacity, and *r* is the intrinsic growth rate of the stock.

#### 3.1.2. Total Allowable Catch

The total allowable catch at a year *y* + 1 is determined with the following harvest control rule:

(2) $$\begin{array}{c} {\text{TAC}}_{y+1}=\frac{aB_{4+,y}+{\text{TAC}}_{y}}{2}, \end{array}$$

where *a* represents harvest rate and *B*_4+,*y*_ is the fishable biomass at year *y* + 1, which consists of cod large enough to be caught [47]. For *a* we used the value 0.2.

### 3.2. Discrete Event Model

The discrete event model simulates fishing trips of three different types of longliners and a bottom trawler. Ideally the model would make use of information from logbooks and use data on trip basis, information such as duration of trip, distance sailed, and amount of catch and oil consumption as an input. In this study only public data on quota allocation and landings were used and scaled over the whole fishing fleet under consideration.

The model outputs are catch numbers, economic performance, and CO_2_ equivalences.

Catch numbers for each vessel are estimated with data over quota allocations published by the Directorate of Fisheries [48].

#### 3.2.1. Economic Impact

**Figure 4:**
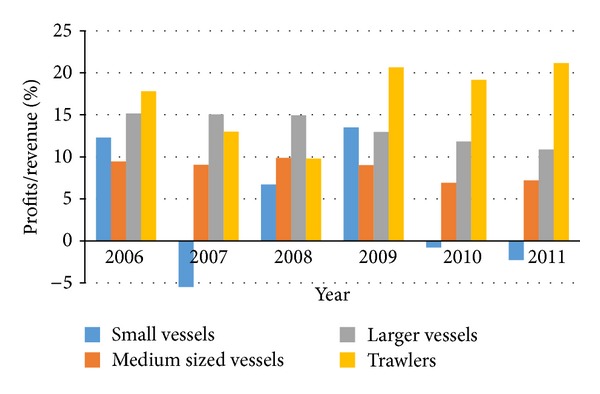
Profits as a ratio of total revenue by vessel type.

Economic performance is measured by multiplying revenue with the ratio of net profit and revenue but this information is available from Statistics Iceland for different vessel types (see (3)).  Figure 4 shows the economic performance of the four different vessels during 2006–2011. This shows clearly how unstable the operating results have been for the small vessels. Average numbers dating back to 1997 were used in the model:

(3) $$\begin{array}{c} \text{Profit}=\text{Value}\;\;\text{of}\;\;\text{fish}\cdot \text{Catch}\cdot \text{PR}, \end{array}$$

where PR = Profit/Net  revenue.

#### 3.2.2. Environmental Impact

The environmental impact of each of the fishing gears was measured in CO_2_ equivalences and based on results from an LCA carried out in 2009. That study showed that one kilo of trawled cod had a 5.14 kg CO_2_ equivalence while a long lined cod is added up to 1.58 kg CO_2_ equivalence [26]. In the same study, it was revealed that the hot spot in the life cycle of cod is the fishing phase.

#### 3.2.3. Social Impact

It is not an easy task to simulate social impact of changing management policies. In this study the only social factor taken into account is number of jobs on each vessel. It might also be relevant to take jobs onshore into account since many of the longliners do not have baiting machines on-board and thus create jobs on land.

### 3.3. Model Validation

The model was validated using available historical data as an input. 

#### 3.3.1. Biological Growth

Stock assessment data from the Icelandic Marine Research Institute was fitted to the logistic model (1) with a linear regression. With 57 data points, the following fit was obtained as shown in Table 1.

**Figure 5:**
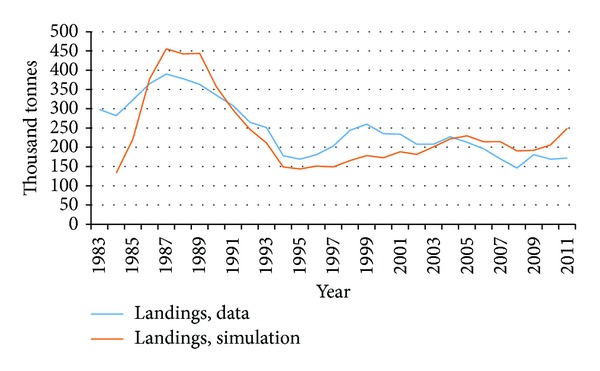
Comparison of output from model simulations and actual stock assessment data for fishable biomass.

**Figure 6:**
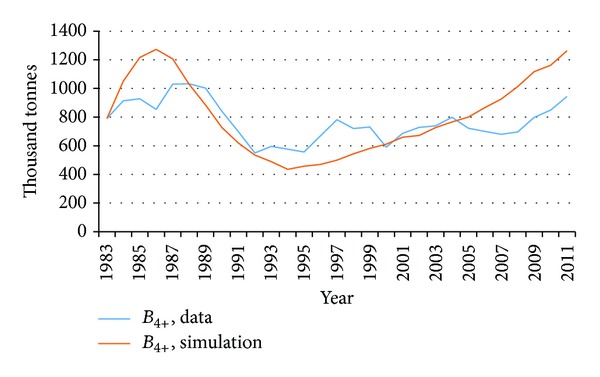
Comparison of output from model simulations and actual data for landings of cod.

These results are not far from results obtained by [49]. Moreover, by running the model with historical catch data as an input, results are shown in Figures 5 and 6. 

There we compare our results from simulation runs with data from 1983. The model gives good results in comparison with data from the mid-eighties until present times which is the period when the demersal stocks of Iceland have been controlled under a quota management scheme and the cod stock has been quite stable. The model however does not account very well for the fluctuations in the stock due to overfishing in the years before the ITQs were imposed. These fluctuations are very visible in the graphs where there is a large gap between the blue and the red line. This we find acceptable as, in the foreseeable future, the stock will with no doubt continue to be controlled with catch quotas, and thus maintain its equilibrium.

Other results such as number of jobs, economic performance, and number of vessels were compared to current numbers for validation purpose when running the model with actual harvest rates from historical data.

## 4. Results

**Figure 7:**
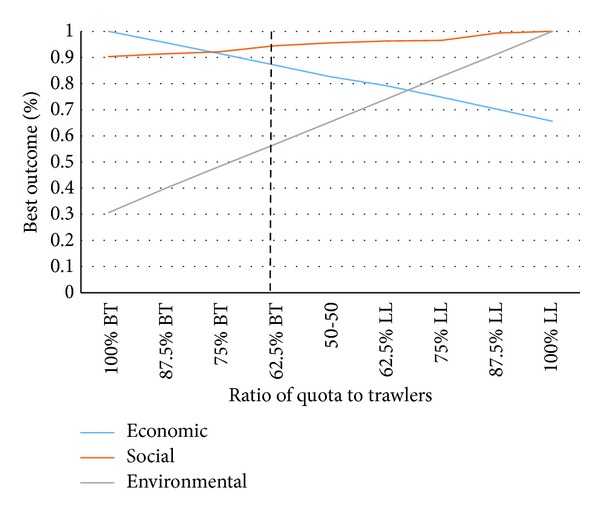
Main results from running simulations with different quota allocations. To the far left we display the case where the entire quota under consideration is allocated to bottom trawlers and the far right shows the opposite case with the entire quota allocated to longliners.

The main objective of the study was to use simulation to determine the optimal ratio of quota allocated to trawlers versus longliners with the multiobjective aim of maximising profit and number of jobs while minimising environmental impact. The model was run multiple times over ten years for different values of *q* which determines division between quota allocated to bottom trawlers and longliners.  Figure 7 shows the results from these runs. The results are displayed in such a way that for each category, each value is displayed as a proportion of the best possible outcome. This is one way of displaying results from the model which are relevant for the current characteristics of these fishing methods.

The best possible economic outcome is obtained when the entire quota is allocated to bottom trawlers whereas the best environmental outcome is at the opposite end, where the entire quota is allocated to longliners. The dashed line in Figure 7 shows the current allocation policy, which leans towards maximizing profitability rather than minimizing environmental impact. If the policy were to lean more towards the intersection of the economic and environmental components, we would get the best possible outcome, assuming that the two components have the same importance. However, the results depend on actual values rather than the potential benefits as well as the subjective weighting of each factor. The model does not take into account jobs in baiting that are created onshore because of longliners.

By expanding the model boundaries, we are likely to see even more positive effects of longliners and a sharper contrast between longliners and bottom trawlers in terms of social aspects. This also leads to a more distribution of wealth which surely would be accounted for as a positive social impact.

## 5. Discussion 

In this paper we have presented the first steps in combining a SD and a DE model resulting in a holistic model of a system while looking at parts of it in more detail. In this study, we used publicly accessible data on landings and quota allocations, which were scaled over the whole fleet under consideration. The output of this work is a simple model which can be improved by adding more system details. Next step is to add more species but the model is easily scalable in terms of number of species. Another obvious step to make in terms of improving the analysis is to expand the system boundaries, for instance to include jobs throughout the whole value chain.

We present a simulation framework which makes it possible to combine LCA data with a hybrid DES-SD model. For obtaining reliable results, the LCA data must be based on a solid ground. Just as the literature on LCA on fisheries underlines, it is of great importance to have access to high quality data on fuel consumption. Using logbook data, as an input to the DE model, the fishing phase could be modelled in more detail. Logbooks include detailed data on fishing activities and this data can be converted to fuel consumption per trip, and this would be of great value since the fishing phase is the part of the life cycle of cod which has the most negative environmental impact due to fossil fuel consumption. With data from logbooks and more detailed operational data, the model could be more realistic and used for further scenario evaluation on quota allocation. Simulation gives the opportunity to move from static results, which the traditional LCA offers to stochastic results, obtained by exploring changes in different factors/policies that might affect the fuel consumption. In terms of future research, it would be possible to model agent based vessels for finding company operations revenue and equilibrium based on different quota allocations. Such a model could be used to identify opportunities to minimise environmental impact and reduce cost by simulating alternative fishing routs for the vessels.

## 6. Conclusions

To conclude, the findings made from the combined SD DE model show and confirm the results in terms of clarification of economic and environmental impact of longliners versus bottom trawlers. The model also shows a need for a larger more complete modelling approach including logbooks from the vessels for increasing accuracy on catch and redirection of traffic to minimize cost and environmental impact while maintaining job opportunities.

## Figures and Tables

**Table 1:** Results from fitting stock data to a logistic model with linear regression.

	Parameter	*t*-statistic
*r*	0.4700	6.6559
*K*	2654.44	2.5561
